# Comparative Study of Paraneoplastic and Nonparaneoplastic Autoimmune Encephalitis With GABA_B_R Antibodies

**DOI:** 10.1212/NXI.0000000000200229

**Published:** 2024-04-24

**Authors:** Florian Lamblin, Jeroen Kerstens, Sergio Muñiz-Castrillo, Alberto Vogrig, David Goncalves, Veronique Rogemond, Geraldine Picard, Marine Villard, Anne-Laurie Pinto, Marleen H. Van Coevorden-Hameete, Marienke A. De Bruijn, Juna M. De Vries, Marco Schreurs, Louise Tyvaert, Lucie Hopes, Jerome Aupy, Cecile Marchal, Dimitri Psimaras, Laurent Kremer, Veronique Bourg, Jean-Christophe G. Antoine, Adrien Wang, Philippe Kahane, Sophie Demeret, Guido Ahle, Vicente Peris Sempere, Noemie Timestit, Mikail Nourredine, Aurelien Maureille, Marie Benaiteau, Bastien Joubert, Emmanuel Mignot, Maarten J. Titulaer, Jerome Honnorat

**Affiliations:** From the French Reference Center on Paraneoplastic Neurological Syndrome and Autoimmune Encephalitis (F.L., V.R., G.P., M.V., A.-L.P., M.B., B.J., J.H.), Hospices Civils de Lyon; Institut MeLiS INSERM U1314/CNRS UMR 5284 (F.L., V.R., G.P., M.V., A.-L.P., M.B., B.J., J.H.), Université Claude Bernard Lyon 1; Department of Neurology (F.L.), University Hospital of La Réunion, Saint-Pierre (La Réunion), France; Department of Neurology (J.K., M.H.V.C.-H., M.A.D.B., J.M.V., M.J.T.), Erasmus Medical Center, Rotterdam, The Netherlands; Stanford Center for Sleep Sciences and Medicine (S.M.-C., V.P.S., E.M.), Stanford University, Palo Alto, CA; Clinical Neurology (A.V.), Department of Neurosciences, Azienda Sanitaria Universitaria Friuli Centrale (ASU FC); Department of Medicine (DAME) (A.V.), University of Udine Medical School, Italy; Department of Immunology (D.G.), Hôpital Lyon Sud, Hospices Civils de Lyon, France; Department of Immunology (M.S.), Laboratory Medical Immunology, Erasmus Medical Center, Rotterdam, The Netherlands; Department of Neurology (L.T., L.H.), University Hospital of Nancy; Department of Clinical Neurosciences (J.A., C.M.), University Hospital of Bordeaux, Bordeaux, France; Department of Neuro-Oncology (D.P.), Pitié Salpêtrière Hospital, AP-HP, Paris; Department of Neurology (L.K.), University Hospital of Strasbourg; Department of Neurology (V.B.), Côte d'Azur University, Nice; Department of Neurology (J.-C.G.A.), University Hospital of Saint-Etienne; Stroke Center Neurology Division (A.W.), Hopital Foch, Suresnes; University Grenoble Alpes (P.K.), Inserm, U1216, CHU Grenoble Alpes, Grenoble Institut Neurosciences; Neurological Intensive Care Unit (S.D.), Pitié-Salpêtrière Hospital, AP-HP, Paris; Department of Neurology (G.A.), Hôpitaux Civils de Colmar; Department of Public Health (N.T., M.N.), Hospices Civils de Lyon; and Department of Medicine (A.M.), Centre Leon Berard, UNICANCER, Lyon, France.

## Abstract

**Background and Objectives:**

While patients with paraneoplastic autoimmune encephalitis (AE) with gamma-aminobutyric-acid B receptor antibodies (GABA_B_R-AE) have poor functional outcomes and high mortality, the prognosis of nonparaneoplastic cases has not been well studied.

**Methods:**

Patients with GABA_B_R-AE from the French and the Dutch Paraneoplastic Neurologic Syndromes Reference Centers databases were retrospectively included and their data collected; the neurologic outcomes of paraneoplastic and nonparaneoplastic cases were compared. Immunoglobulin G (IgG) isotyping and human leukocyte antigen (HLA) genotyping were performed in patients with available samples.

**Results:**

A total of 111 patients (44/111 [40%] women) were enrolled, including 84 of 111 (76%) paraneoplastic and 18 of 111 (16%) nonparaneoplastic cases (cancer status was undetermined for 9 patients). Patients presented with seizures (88/111 [79%]), cognitive impairment (54/111 [49%]), and/or behavioral disorders (34/111 [31%]), and 54 of 111 (50%) were admitted in intensive care unit (ICU). Nonparaneoplastic patients were significantly younger (median age 54 years [range 19–88] vs 67 years [range 50–85] for paraneoplastic cases, *p* < 0.001) and showed a different demographic distribution. Nonparaneoplastic patients more often had CSF pleocytosis (17/17 [100%] vs 58/78 [74%], *p* = 0.02), were almost never associated with KTCD16-abs (1/16 [6%] vs 61/70 [87%], *p* < 0.001), and were more frequently treated with second-line immunotherapy (11/18 [61%] vs 18/82 [22%], *p* = 0.003). However, no difference of IgG subclass or HLA association was observed, although sample size was small (10 and 26 patients, respectively). After treatment, neurologic outcome was favorable (mRS ≤2) for 13 of 16 (81%) nonparaneoplastic and 37 of 84 (48%) paraneoplastic cases (*p* = 0.03), while 3 of 18 (17%) and 42 of 83 (51%) patients had died at last follow-up (*p* = 0.008), respectively. Neurologic outcome no longer differed after adjustment for confounding factors but seemed to be negatively associated with increased age and ICU admission. A better survival was associated with nonparaneoplastic cases, a younger age, and the use of immunosuppressive drugs.

**Discussion:**

Nonparaneoplastic GABA_B_R-AE involved younger patients without associated KCTD16-abs and carried better neurologic and vital prognoses than paraneoplastic GABA_B_R-AE, which might be due to a more intensive treatment strategy. A better understanding of immunologic mechanisms underlying both forms is needed.

## Introduction

Autoimmune encephalitis (AE) associated with gamma-aminobutyric-acid B receptor antibodies (GABA_B_R-AE) was first described in 2010 but remains a rare disease with a potentially devastating vital and functional prognosis.^1-4^ The clinical feature usually consists of a limbic encephalitis (LE), with antiseizure medication-resistant epileptic seizures, confusion, and anterograde amnesia, but it can also present with rapidly progressive dementia without prominent seizures.^2,3^ Approximately 50%–60% of patients have an underlying malignancy, most commonly small cell lung cancer (SCLC) in elderly smoker men, and generally neurologic symptoms precede and ultimately lead to the tumor diagnosis.^4,5^ Despite increasing knowledge on some pathophysiologic features of GABA_B_R-AE,^6,7^ data regarding long-term outcomes are still scarce.^3,4^ Although the functional outcome is mainly driven by the neurologic syndrome and its medical management, including immunotherapy delay or occurrence of intensive care unit (ICU) complications, the survival is also determined by the frequent presence of an underlying malignancy.^8-10^ Nevertheless, whether patients with SCLC and nonparaneoplastic cases differ in clinical onset and long-term prognosis has not been studied in detail yet.^3,11,12^ In this study, we aimed to compare the clinical presentation, immunogenetic characteristics, and neurologic outcome of paraneoplastic and nonparaneoplastic GABA_B_R-AE identified in the French and the Dutch Paraneoplastic Neurologic Syndromes Reference Centers between 2011 and 2022.

## Methods

### Study Design and Patient Selection

We retrospectively included patients diagnosed with GABA_B_R-AE from January 2011 to June 2022 at 2 European Reference Network sites (ERN-RITA) for Paraneoplastic Neurologic Syndromes (PNS): the French Reference Center in Lyon (France) and the Dutch Reference Center in Rotterdam (The Netherlands). GABA_B_R antibodies (GABA_B_R-abs) were identified in CSF and/or serum by immunohistochemistry/immunohistofluorescence using commercial and/or in-house cell-based assay (CBA), using either HEK293 or CHO cells expressing GABA_B_1a and GABA_B_2 subunits as previously described.^2,3^ In addition, antibodies against potassium channel tetramerization domain containing 16 (KCTD16-abs) were identified in CSF and/or serum with in-house CBA, as described elsewhere.^3^ This observational retrospective multicentric cohort study is reported following the Strengthening the Reporting of Observational Studies in Epidemiology (STROBE) guidelines.^13^

### Data Collection

Data on clinical presentation, treatment, and results of ancillary investigations (EEG, MRI, CSF findings, presence of coexisting antibodies including KCTD16-abs in serum and/or CSF, and oncological investigations) were retrospectively collected by independent physicians in each reference center (F.L., J.K., M.C.-H., M.d.B., and J.d.V.) from medical reports obtained at the time of diagnosis and by request to patients' treating physicians at the time of the study. Diagnostic criteria by Graus et al. were applied to confirm the diagnosis of AE.^14,15^

### Primary and Secondary Outcomes

We compared the initial characteristics, treatment, neurologic outcome, and survival of paraneoplastic and nonparaneoplastic cases, only in patients for whom the presence of a cancer was confirmed or considered to have been sufficiently explored. Duration of follow-up was defined from symptom onset to the last medical visit or death. A favorable neurologic outcome was considered as a modified Rankin scale (mRS) ≤ 2 after immunotherapy and/or oncological treatment, whereas neurologic improvement was defined as an mRS improvement of ≥1 point. An episode of relapse was defined as a new onset or worsening of encephalitic symptoms after an initial improvement or stabilization of at least 2 months.

### Immunoglobulin G Isotypes and Human Leukocyte Antigen Genotyping

In a subset of French patients for whom samples were available, immunoglobulin G (IgG) isotyping and human leukocyte antigen (HLA) genotyping were performed. IgG isotyping was performed using mouse anti-human antibodies that specifically recognize IgG1 or IgG4, as previously described.^16^ Genotypes at four-digit resolution for HLA class I (loci A, B, and C) and class II (loci DPA1, DPB1, DQA1, DQB1, DRB1, DRB3, DRB4, and DRB5) were imputed from available genome-wide association data (GWAS); allele carrier frequencies were then compared between patients (entire cohort and according to cancer status) and controls (239 healthy participants provided by the Stanford Center for Sleep Sciences and Medicine) using logistic regression controlled by principal component analysis; multiple comparisons were corrected by the Bonferroni method, and corrected *p* < 0.05 were considered statistically significant.^17^ HLA analysis was performed with R software.

### Statistical Analysis

Quantitative variables are reported as mean (SD) or median (range or interquartile range), while qualitative variables are reported as number (percentage). Paraneoplastic and nonparaneoplastic cases were compared using the Fisher exact test and the Student t-test. Neurologic outcome and survival were compared among the 2 groups using logistical regression and Kaplan-Meier curves, respectively, and were secondary adjusted for confounding variables (age, sex, immunosuppressive drugs [including second-line immunotherapy or chemotherapy], and ICU admission) using multivariate logistical regression and Cox model, respectively. Statistical significance was established as *p* < 0.05. Statistical analyses were performed using jamovi^18^ and R version 4.0.3 (R Foundation for Statistical Computing, Vienna, Austria).

### Standard Protocol Approvals, Registrations, and Patient Consents

Approval from the ethics committee of the Hospices Civils de Lyon (IRB00013204) was obtained. Informed consent was not required, but information about the study was transmitted to the patients or their physician.

### Data Availability

Patient-related data will be shared on reasonable request from any qualified investigator, maintaining anonymization of the individual patients.

## Results

### General Cohort

**Figure 1:**
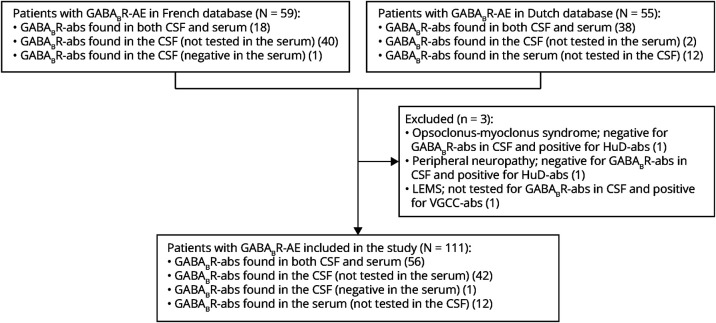
Flowchart LEMS = Lambert-Eaton myasthenic syndrome.

We retrospectively identified 114 patients with GABA_B_R-abs in the French and Dutch databases and finally included 111 patients in the analysis (Figure 1); among them, a total of 54 patients (22 French and 32 Dutch) have previously been reported.^2,3^ Three patients were excluded because GABA_B_R-abs were doubtful and suggested by only 1 CBA test in the serum. Immunofluorescences were negative, and the CSF was negative for 2 patients and unavailable for the last one. We finally considered this CBA testing in the serum as false positive results; the clinical pictures of these 3 patients were clearly explained by the presence of another autoantibody (Hu-abs in 2 patients and VGCC-abs in 1). The main clinical and paraclinical characteristics of the 111 included patients are presented in Table 1. Median age at onset was 66 years (range 19–88), and 44 of 111 (40%) were women. Seizures were the first symptom at disease onset in 88 of 111 (79%) patients; 55 of 88 (63%) had generalized tonic-clonic seizures, 11 of 88 (13%) had focal seizures, and 22 of 88 (25%) had both types of seizures. At onset, seizures were isolated in 51 of 88 (58%) patients and accompanied by cognitive impairment and/or behavioral disorders in 37 of 88 (42%) patients. The remaining 23 of 111 (21%) patients without seizures at disease onset (19/23 who still developed seizures later in the disease course) presented with rapidly progressive cognitive decline and behavioral disorders (13/111, 12%), isolated rapidly progressive cognitive decline (9/111, 8%), or isolated behavioral disorders (1/111, 1%). Furthermore, at disease onset, 54 of 108 (50%) patients were admitted in an ICU due to status epilepticus (41/52, 79%), unexplained low level of consciousness (7/52, 14%), and/or respiratory or hemodynamic failure (5/52, 10%). Over the whole course of the disease, 107 of 111 (96%) patients had seizures, 80 of 111 (72%) had anterograde amnesia, 32 of 111 (29%) had language disorders, 57 of 111 (51%) had behavioral disorders, and 17 of 111 (15%) had mood disorders.

**Table 1:** Patients' General Characteristics

Variables	All patients	Paraneoplastic	Nonparaneoplastic	*p* Value
Number of patients	111	84/111 (76)	18/111 (16)	
Female, n (%)	44/111 (40)	34/84 (41)	7/18 (39)	0.90
Age at onset (y), median (IQR, range)	67 (61–72, 19–88)	67 (62–71, 50–85)	54 (43–69, 19–88)	<0.001
Smoker	79/100 (79)	65/77 (84)	9/17 (53)	0.004
Clinical characteristics at onset				
Prodromal symptoms	15/108 (14)	12/82 (15)	1/18 (6)	0.45
Acute or subacute onset	102/111 (92)	77/84 (92)	17/18 (94)	1
Limbic encephalitis	105/111 (95)	81/84 (96)	15/18 (83)	0.07
Seizures	88/111 (79)	69/84 (82)	14/18 (78)	0.67
Cognitive impairment	54/111 (49)	36/84 (43)	10/18 (56)	0.33
Behavioral disorders	34/111 (31)	22/84 (26)	8/18 (44)	0.13
ICU admission	54/108 (50)	42/82 (51)	6/18 (33)	0.17
Antineuronal antibodies				
GABA_B_R-abs	111/111 (100)	84/84 (100)	18/18 (100)	NA
Serum	68/69 (99)	42/43 (98)	18/18 (100)	0.52
CSF	99/99 (100)	76/76 (100)	16/16 (100)	NA
KCTD16-abs	68/93 (73)	61/70 (87)	1/16 (6)	<0.001
Serum	68/69 (99)	46/54 (85)	1/13 (8)	<0.001
CSF	98/98 (100)	44/53 (83)	1/13 (8)	<0.001
Other antineuronal antibody	28/111 (25)	25/84 (30)	1/18 (6)	0.09
CSF characteristics				
Delay from onset (d), median (IQR, range)	12 (4–31, –6-365)	11 (3–26, –6-160)	22 (6–36, 0–365)	0.03
Normal	11/107 (11)	7/79 (9)	0/17 (0)	0.35
Pleocytosis (>5 cells/mm^3^)	82/102 (80)	58/78 (74)	17/17 (100)	0.02
Cell count (cells/mm^3^), median (IQR, range)	14 (5–37, 0–296)	11 (5–31, 0–296)	22 (13–43, 6–150)	0.51
Elevated protein (>50 mg/dL)	50/95 (53)	42/72 (58)	6/17 (35)	0.11
Protein level (mg/dL), median (IQR, range)	51 (40–76, 19–425)	51 (40–80, 21–425)	40 (33–58, 19–121)	0.20
Oligoclonal bands	26/31 (86)	21/23 (91)	3/5 (60)	0.14
MRI characteristics				
Delay from onset (d), median (IQR, range)	10 (5–23, –2-220)	8 (5–19, –2-172)	11 (4–35, 0–220)	0.16
Normal	47/101 (46)	36/78 (46)	9/18 (50)	0.80
Mesiotemporal T2/FLAIR hyperintensities	44/101 (44)	36/78 (46)	7/18 (39)	0.61
EEG characteristics				
Delay from onset (d), median (IQR, range)	7 (2–16, 0–469)	6 (2–12, 0–469)	10 (3–23, 0–153)	0.99
Normal	30/95 (32)	26/72 (36)	2/16 (13)	0.08
Epileptic features	37/95 (39)	24/72 (33)	9/16 (56)	0.10
Encephalopathic features	37/91 (41)	30/70 (43)	6/16 (38)	0.78
Immunotherapy				
Delay from onset (d), median (IQR, range)	27 (16–43, 3–439)	25 (16–43, 6–411)	34 (20–130, 11–439)	0.02
First-line immunotherapy	95/108 (88)	75/82 (92)	17/18 (94)	1
Second-line immunotherapy	29/108 (27)	18/82 (22)	11/18 (61)	0.003
Outcomes				
Length of follow-up (mo), median (IQR, range)	10 (3–21, 0–111)	10 (5–17, 0–111)	22 (7–37, 3–61)	0.03
Favorable outcome (lowest posttreatment mRS ≤2)	50/97 (52)	37/77 (48)	13/16 (81)	0.03
Relapse	10/71 (14)	8/54 (15)	2/14 (14)	0.96
Death	54/111 (49)	42/83 (51)	3/18 (17)	0.008

Abbreviations: ICU = intensive care unit, IQR = interquartile range, mRS = modified-Rankin Scale.

### Cancer Associations

A total of 71 of 111 (64%) patients were diagnosed with cancer; 64 had SCLC (90%), the remaining ones presenting with lepidic pulmonary adenocarcinoma, poorly differentiated lung adenocarcinoma, small cell neuroendocrine prostatic cancer, neuroendocrine rectal cancer, neuroendocrine medullary thyroid carcinoma, pancreatic adenocarcinoma, and malignant thymoma (one each, 1%); all of them were considered paraneoplastic cases. Furthermore, 13 of 111 patients (12%) had a diagnosis of probable lung cancer because a lung and/or mediastinal mass suspicious of malignancy was identified on a thoracic CT scan and/or PET scan. These patients also presented asthenia and loss of weight but did not reach a final tissue-based diagnosis; 9 patients died before a definitive diagnosis could be made, while 4 patients were still alive at last follow-up (only initial data without follow-up for 1 patient; 5 months, 7 months, and 8 months of follow-up for the 3 others). Among them, 1 patient had a biopsy failure and 3 patients had unconclusive biopsies. These 13 patients were also classified as paraneoplastic cases. Conversely, 18 of 111 patients (16%) were diagnosed as nonparaneoplastic GABA_B_R-AE because there was no evidence of an underlying cancer after thorough screening, which in all but one case included a PET scan and/or a full-body CT scan, and a median (interquartile range [IQR]) follow-up of 22 (7–37) months; the remaining patient died, and no evidence of cancer was found during autopsy. Nine patients (9/111, 8%) could not be classified because of insufficient tumor screening or missing data.

### Treatment and Outcomes

First-line immunotherapy (IV immunoglobulins, steroids, and/or plasma exchange) was initiated with a median (IQR) of 27 (16–43) days after disease onset in 95 of 108 patients (88%). In addition, after a median of 87 days (IQR 53–229), 29 of 108 patients (27%) received second-line immunotherapy: rituximab in 17 of 108 (16%), cyclophosphamide in 7 of 108 (7%), and both in 5 of 108 (5%). Oncological treatments of patients with an associated tumor are reported in Table 2. After immunotherapy and/or oncological treatment, 71 of 108 patients (66%) improved neurologically. During the course of the disease, 10 of 71 patients (14%) relapsed after an initial improvement, while 54 of 111 patients (49%) finally died. Of note, 2 patients received a cancer immunotherapy after the diagnosis of GABA_B_R-AE (respectively, 4 and 6 cycles) without neurologic relapse.

**Table 2:** Characteristics of Tumor Diagnosis and Oncological Treatments

Variables	All patients
Number of patients	111
Tumor diagnosis	
Delay from onset (d), median (IQR, range)	42 (18–79, –719-825)
Tumor with histologic evidence	71/111 (64)
SCLC	64/71 (90)
Other tumor	7/71 (10)
Probable tumor without histologic evidence	13/111 (12)
No tumor with extensive screening	18/111 (16)
Undetermined tumoral status	9/111 (8)
Oncological treatment	
Chemotherapy	59/107 (55)
Immunotherapy	2/105 (2)
Surgery	4/105 (4)
Radiotherapy	22/101 (22)
No treatment	36/107 (34)

Abbreviations: IQR = interquartile range, SCLC = small cell lung cancer.

### Comparison of Paraneoplastic and Nonparaneoplastic Cases

**Figure 2:**
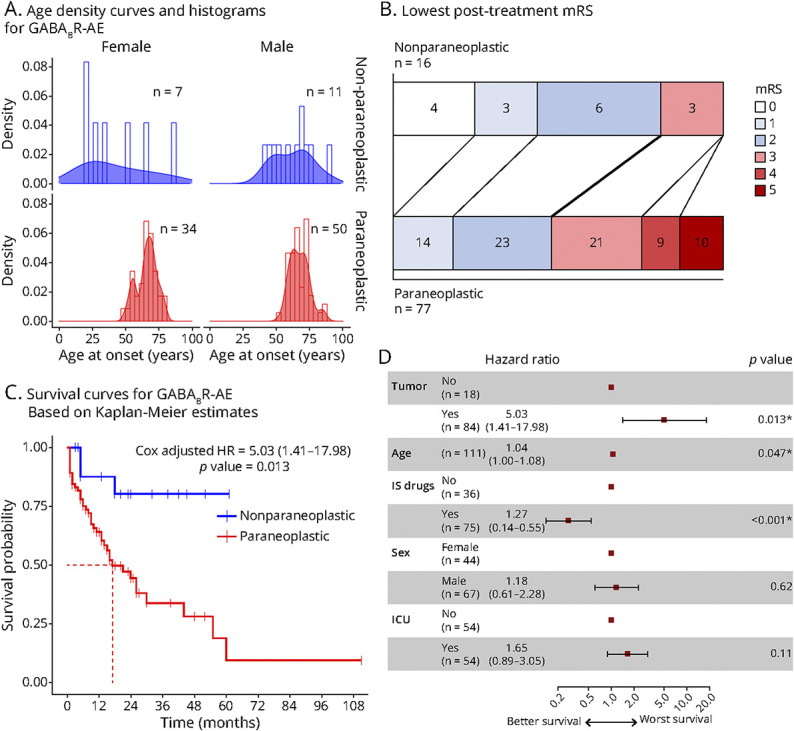
Clinical Course Comparison of Paraneoplastic and Nonparaneoplastic GABA_B_R-AE IS = immunosuppressive, mRS = modified-Rankin scale. (A) Nonparaneoplastic forms of GABA_B_R-AE occur in young adult women or middle-aged to elderly men, while paraneoplastic forms have a single incidence peak in the seventh decade of both sexes. After treatment, nonparaneoplastic cases evolve toward a better neurologic status (B) and have a better survival rate (C) within log-rank univariate analysis (*p* = 0.0048, data not shown) and Cox model multivariate analysis adjusted for confounding factors. Absence of tumor, lower age, and immunosuppressive drugs (second-line immunotherapy or chemotherapy) are associated with a better survival (D).

We then compared the characteristics and neurologic outcome of the 84 paraneoplastic and 18 nonparaneoplastic cases (Table 1 and Figure 2). Nonparaneoplastic patients were younger, with a median age of 54 years (range 18–88) compared with 67 (50–85) in the paraneoplastic group (*p* < 0.001). The nonparaneoplastic group showed a different demographic distribution with, in addition to the usual incidence peak occurring in older patients, few cases in young adult women and in middle-aged men (Figure 2A). Furthermore, nonparaneoplastic cases were less likely to smoke than paraneoplastic cases (9/17 [53%] vs 65/77 [84%], *p* = 0.004; Table 1) and more often had CSF pleocytosis (17/17 [100%] vs 58/78 [74%], *p* = 0.02), while this finding may also reflect some form of bias, as nonparaneoplastic cases without CSF pleocytosis might have been missed because antibody testing was not considered in these patients. The remaining clinical and paraclinical features at onset were similar between both groups (Table 1; the detailed characteristics of nonparaneoplastic cases are further described in Table 3); however, nonparaneoplastic patients were more often treated with a second-line immunotherapy than paraneoplastic ones (11/18 [61%] vs 18/82 [22%], *p* = 0.003). Similarly, although the maximum mRS before treatment did not differ between paraneoplastic and nonparaneoplastic patients, nonparaneoplastic cases were more likely to evolve toward a favorable neurologic outcome after immunotherapy than paraneoplastic patients (13/16 [81%] vs 37/77 [48%], *p* = 0.03; OR = 4.70, 95% CI 1.24–17.86, *p* = 0.012; Table 1 and Figure 2). This difference was no longer statistically significant after adjustment for confounding factors (OR = 3.32, 95% CI 0.67–20.83, *p* = 0.15) because increased age (per unit increase OR = 0.91, 95% CI 0.85–0.97, *p* = 0.002) and ICU admission (OR = 0.23, 95% CI 0.08–0.61 *p* = 0.003; Table 4) were negatively associated with a favorable neurologic outcome. At last follow-up, the survival rate was significantly worse for paraneoplastic cases of whom 42 of 83 (51%) were dead vs 3 of 18 (17%) in the nonparaneoplastic group (*p* = 0.008; Table 3). Median of the overall survival was 17 months (95% CI 13–44) for paraneoplastic cases while incalculable for the nonparaneoplastic group due to the low mortality rate (Figure 2C). After adjustment for confounding factors, the survival of paraneoplastic cases remained significantly worse (HR = 5.03, 95% CI 1.41–17.98, *p* = 0.013); the use of immunosuppressive drugs (HR = 3.70, 95% CI 1.82–7.14, *p* < 0.001) was associated with a better survival, while increased age (per unit increase HR = 1.04, 95% CI 1.00–1.08, *p* = 0.047) was associated with a worse survival.

**Table 3:** Detailed Characteristics of Nonparaneoplastic Patients

N	Age smoker	Clinical data at onset	Second symptoms (delay in days)	ICU (LOS in days)	First paraclinical data (delay from onset in days) and oncological investigations	GABA_B_R-abs	KCTD16-abs	Worst pretreatment mRS (delay from onset in d)	Immunotherapy (delay from onset in days)	Best posttreatment mRS (time of achievement from onset in months)	LOF in months
1	<50 y No	FS and GTCS	SRSE (9)	Y (43)	EEG (10) = encephalopathic, MRI (10) = normal, CSF (9) = 35 WBC/mm^3^, no elevated protein, OCB NA PET scan	Positive in CSF and serum	NA	5 (9)	Steroids and PE (11), then RTX (26)	0 (3)	24 (alive)
2	<50 y No	FS and behavioral disorders	Generalized SE (10)	N	EEG (0) = diffuse spikes, MRI (0) = normal, CSF (0) = 90 WBC/mm^3^, no elevated protein, OCB NA PET scan	Positive in CSF and serum	Negative in CSF and serum	3 (10)	IVIg (19), then CP (53) and RTX (59)	1 (12)	13 (alive)
3	<50 y No	GTCS	RPCC and behavioral disorders (1)	N	EEG (0) = temporal discharges, MRI (0) = normal, CSF (365) = 5 WBC/mm^3^, no elevated protein, OCB present PET scan	Positive in CSF and serum	Negative in CSF, NA in serum	3 (15)	IVIg (439) and steroids (444), then RTX (1665)	0 (60)	61 (alive)
4	<50 y No	GTCS	Cognitive and behavioral disorders (5)	N	EEG (5) = encephalopathic, MRI (5) = normal, CSF (8) = 150 WBC/mm^3^, no elevated protein, no OCB PET scan and CT scan	Positive in CSF and serum	Negative in CSF and serum	4 (5)	IVIg (23) and steroids (23), then RTX (327)	2 (6)	18 (alive)
5	<50 y No	Temporal FS	GTCS, cognitive and behavioral disorders (30)	N	EEG (1) = temporal slowing focus, MRI (4) = bilateral mesiotemporal T2/FLAIR HI, CSF (36) = 14 WBC/mm^3^, no elevated protein, OCB NA PET scan and CT scan	Positive in CSF and serum	Negative in CSF and serum	4 (30)	IVIg (38) and steroids (43)	0 (5)	5 (alive)
6	<50 y No	GTCS	FS, cognitive and behavioral disorders (15)	N	EEG (36) = normal, MRI (24) = normal, CSF (22) = 22 WBC/mm^3^, elevated protein (54 mg/dL), OCB NA PET scan and CT scan	NA in CSF, positive in serum	NA in CSF, negative in serum	4 (15)	Steroids (NA)	2 (12)	23 (alive)
7	≥50 y Yes	GTCS	SRSE (204)	Y (11)	EEG (153) = spikes and slowing focus, MRI (220) = normal, CSF (204) = 37 WBC/mm^3^, elevated protein (60 mg/dL), OCB NA PET scan and CT scan	Positive in CSF and serum	NA in CSF, negative in serum	5 (204)	IVIg (280), then RTX (385)	2 (12)	32 (alive)
8	≥50 y Yes	FS	SRSE (5)	Y (35)	EEG (6) = sedation, MRI (7) = normal, CSF (6) = 43 WBC/mm^3^, no elevated protein, OCB NA PET scan and CT scan	Positive in CSF and serum	Negative in CSF and serum	5 (5)	IVIg (34)	0 (3)	45 (alive)
9	≥50 y Yes	Focal to GTCS	RPCC and behavioral disorders (<15)	N	EEG (10) = epileptic and encephalopathic features, MRI (11) = normal, CSF (13) = 39 WBC/mm^3^, no elevated protein, OCB NA PET scan and CT scan	Positive in CSF and serum	Negative in CSF and serum	4 (15)	IVIg (20) and steroids (20)	1 (6)	38 (alive)
10	≥50 y No	RPCC and behavioral disorders	None	N	EEG (22) = normal, MRI (22) = mesiotemporal T2/FLAIR HI, CSF (22) = 105 WBC/mm^3^, elevated protein (107 mg/dL), OCB present PET scan	Positive in CSF and serum	Negative in CSF and serum	3 (15)	IVIg (30) and steroids (30), then RTX (51)	3 (NA)	52 (alive)
11	≥50 y Yes	Focal to GTCS	RPCC and behavioral disorders (<15)	N	EEG NA, MRI (3) = mesiotemporal T2/FLAIR HI, CSF (0) = 75 WBC/mm^3^, no elevated protein, OCB NA PET scan and CT scan	Positive in CSF and serum	Negative in CSF and serum	3 (15)	Steroids (2) and IVIg (226), then CP (376)	2 (3)	32 (alive)
12	≥50 y Yes	Temporal FS	Progressive cognitive decline (2 y)	N	EEG (6) = temporal discharges and slowing focus, MRI (5) = right mesiotemporal T2/FLAIR HI, CSF (5) = 6 WBC/mm^3^, elevated protein (121 mg/dL), OCB NA PET scan	Positive in CSF and serum	Negative in CSF, NA in serum	3 (15)	IVIg (231), then CP (470) and RTX (427)	2 (12)	42 (alive)
13	≥50 y No	Temporal FS	Generalized SE (37)	Y (1)	EEG (0) = normal, MRI (0) = normal, CSF (0) = 6 WBC/mm^3^, no elevated protein, no OCB PET scan	Positive in CSF and serum	Negative in CSF, NA in serum	5 (37)	IVIg (33) and steroids (33)	1 (6)	20 (alive)
14	≥50 y Yes	RPCC and behavioral disorders	GTCS (32)	Y (NA)	EEG (NA) = epileptic and encephalopathic features, MRI (82) = no mesiotemporal T2/FLAIR HI, CSF (81) = 11 WBC/mm^3^, no elevated protein, OCB NA PET scan and CT scan	Positive in CSF and serum	Negative in CSF and serum	5 (NA)	IVIg (96) and steroids (96), then RTX (110)	NA	5 (dead)
15	≥50 y NA	RPCC and behavioral disorders	Seizures (NA)	N	EEG (45) = normal, MRI (45) = mesiotemporal T2/FLAIR HI, CSF (45) = 15 WBC/mm^3^, no elevated protein, OCB NA Autopsy	Positive in CSF and serum	Positive in CSF and serum (no cancer evidence at autopsy)	5 (NA)	None (only symptomatic treatments)	NA	5 (dead)
16	≥50 y Yes	GTCS and confusion	SRSE (22)	Y (2)	EEG (11) = spikes, MRI (39) = mesiotemporal T2/FLAIR HI, CSF (24) = 13 WBC/mm^3^, elevated protein (88 mg/dL), OCB present PET scan and CT scan	Positive in CSF and serum	NA in CSF, negative in serum	5 (30)	Steroids (405)	2 (14)	18 (dead)
17	≥50 y Yes	Progressive confusion and psychosis	None	N	EEG NA, MRI (14) = no mesiotemporal T2/FLAIR HI, CSF NA CT scan	NA in CSF, positive in serum	Negative in CSF and serum	3 (15)	IVIg (15) and steroids (15), then CP (NA) and RTX (NA)	3 (3)	3 (alive)
18	≥50 y Yes	GTCS	RPCC (24)	N	EEG (24) = spikes, MRI (42) = bilateral mesiotemporal T2/FLAIR HI, CSF (28) = 16 WBC/mm^3^, elevated protein (58 mg/dL), OCB NA PET scan and CT scan	Positive in CSF and serum	NA	4 (24)	IVIg (56) and steroids (42), then RTX (91)	3 (4)	4 (alive)

Abbreviations: CP = cyclophosphamide, IVIg = IV immunoglobulin, GTCS = generalized tonic-clonic seizures, HI = hyperintensity, LOF = length of follow-up, LOS = length of stay, NA = not available, OCB = oligoclonal bands, PE = plasmatic exchanges, FS = focal seizures, RPCC = rapidly progressive cognitive decline, RTX = rituximab, SRSE = super-refractory status epilepticus, WBC = white blood cell.

**Table 4:** Favorable Neurologic Outcome (mRS ≤2) Comparison Between Paraneoplastic and Nonparaneoplastic Cases of GABA_B_R-AE: Logistical Regression Model Adjusted for Confounding Factors

Variable	OR	Lower 95% CI	Upper 95% CI	*p* Value
Diagnosis of cancer (nonparaneoplastic)	0.30	0.05	1.49	0.15
Age at onset of disease per unit increase	0.91	0.85	0.97	0.002
Immunosuppressive drugs (yes)	1.90	0.61	6.29	0.27
Sex (male)	0.74	0.27	2.02	0.55
ICU admission (yes)	0.23	0.08	0.61	0.003

### Immunologic and Genetic Characteristics

**Figure 3:**
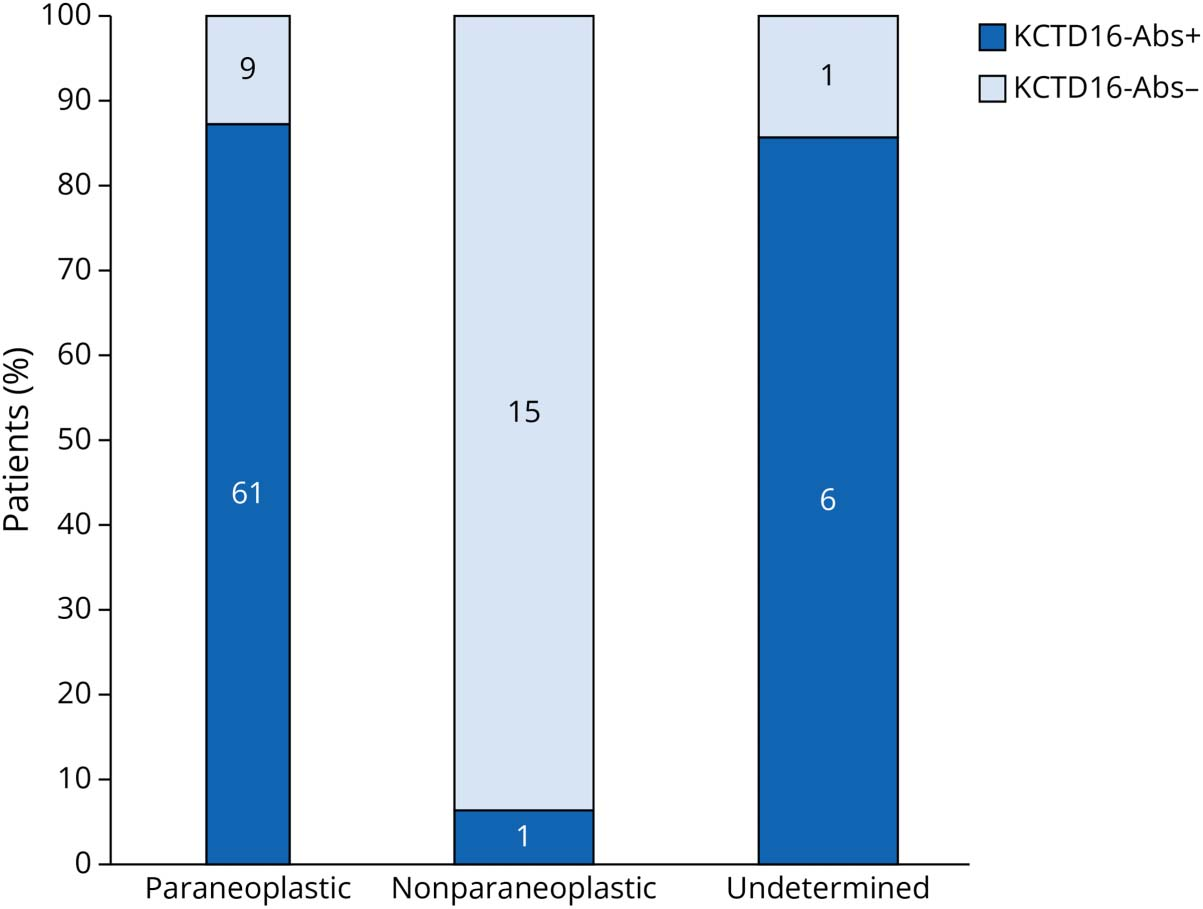
Bar Diagram Showing the Association of Paraneoplastic Cases With KCTD16-abs Patients for which tumoral status could not be determined seem to have the same strong association with KCTD16-abs as paraneoplastic cases, suggesting an unfound malignancy.

KCTD16-abs were found in the CSF and/or serum of 68 of 93 patients (73%) and were significantly more often present in paraneoplastic cases (61/70 [87%] vs 1/16 [6%], *p* < 0.001, Table 1 and Figure 3). KCTD16-abs had a sensitivity of 87%, a specificity of 94%, a positive predictive value of 98%, and a negative predictive value of 63% to detect a paraneoplastic origin. One or more other neuronal antibodies were found in 28 of 111 patients (25%) (12 SOX1-abs, 6 Hu-abs, 5 VGCC-abs, 2 GABA_A_R-abs, 2 AMPAR-abs, 2 amphiphysin-abs, 2 ZIC4-abs, 2 CV2/CRMP5-abs, 1 Ri/NOVA1-abs, and 1 high titer GAD65-abs), mostly in paraneoplastic cases (28/84 [33%] vs 2/18 [11%] in the nonparaneoplastic group, *p* = 0.09, see Table 1).

IgG subclasses 1 and 4 for GABA_B_R-abs were analyzed in 10 patients, and all samples were positive for IgG1 and negative for IgG4 (7 nonparaneoplastic and 3 paraneoplastic; 8 in CSF and 2 in serum).

The HLA genotyping was available for 26 patients (21 paraneoplastic, 5 nonparaneoplastic). No differences in carrier frequencies were observed in controls (n = 239) vs all patients, or between a subset of GWAS principal component (ethnically) matched controls (n = 48) vs the nonparaneoplastic group (data not shown). In paraneoplastic cases, DRB3*03:01 was more frequent in patients (7/21, 33%) vs matched controls (22/191, 12%) (OR = 3.8, 95% IC 1.3–10.9, uncorrected *p* = 0.01, corrected *p* = 0.03), a difference that disappeared after controlling for DRB1*13:02 (7/19, 37% in cases, vs 22/191, 12% in controls; OR = 4.4, 95% CI 1.5–13.1, uncorrected *p* = 0.006, corrected *p* = 0.2), a DRB1 allele in strong linkage disequilibrium with DRB3*03:01. Of interest, 5 of 13 (38%) nonparaneoplastic cases were not of European descent (3 Asians, 1 black African, and 1 Arab/Berber descendants) in contrast to none of the paraneoplastic or undetermined patients (eFigure for French patients, data not shown for Dutch patients).

## Discussion

In this study, the characteristics and prognosis of patients with paraneoplastic and nonparaneoplastic GABA_B_R-AE were investigated. We first showed that nonparaneoplastic forms of GABA_B_R-AE can affect younger patients and seem to have better neurologic outcome after immunotherapy. We then confirmed KCTD16-abs as a strong biomarker of an underlying SCLC, as previously described^3^; KCTD16-abs should therefore be requested in all cases of GABA_B_R-AE without a known tumor, and their presence should warrant thorough and repeated tumor screening.

Although there was no difference between groups regarding the disease severity, nonparaneoplastic cases were more likely to receive second-line immunotherapy; this may reflect a tendency to use more aggressive treatments in younger patients with no oncological comorbidity or to avoid second-line immunotherapy in patients with cancer receiving chemotherapy, because of the risk of drug interactions leading to increased toxicity. Adjusted analyses on confounding factors suggested that a younger age and a more intensive treatment strategy (i.e., immunosuppressive drugs as second-line immunotherapy or chemotherapy) were associated with a better survival, arguing toward the use of immunosuppressive treatments in both paraneoplastic and nonparaneoplastic cases of GABA_B_R-AE. ICU admission was also found to be associated with a worse neurologic outcome, which may be explained by the occurrence of ICU complications, known to be strongly associated with worse long-term functional outcomes of patients with an AE, more than the individual AE subtype.^10^

Of interest, we found 2 different demographic profiles in the nonparaneoplastic group: young nonsmoking women and older smoking patients, the latter were similar to typical paraneoplastic GABA_B_R-AE patients. In theory, even after extensive screening and long follow-up, 9 elderly patients with a history of smoking in the nonparaneoplastic group could have still undetected microscopic or regressive tumors, kept in check by the exaggerated immune response known to be associated with PNS.^6,19-21^ However, the absence of KCTD16-abs in these patients argues against this hypothesis. Among the nonparaneoplastic group, 5 of 18 patients had finally a duration of follow-up of only 3–5 months that might be insufficient to clearly exclude a paraneoplastic origin because many PNS may precede tumor diagnosis of several months to years.^15^ Furthermore, the lack of an obvious environmental trigger in nonparaneoplastic GABA_B_R-AE could suggest the existence of a genetic predisposition. However, we did not find any HLA association, although the number of patients included in the HLA genotyping was very low. Notably, all patients (paraneoplastic and nonparaneoplastic) had GABA_B_R-abs of IgG1 subtype. These findings fit with previously published data on AE with IgG1 antibodies (e.g., NMDAR-AE)^22^ and on PNS (e.g., Hu- or Yo-PNS),^23^ which are not found to be strongly associated with specific HLA genes, in contrast to AE with antibodies of predominantly IgG4 subclass such as LGI1-AE with HLA-DRB1*07:01 and CASPR2-LE with HLA-DRB1*11:01.^24−26^

Of note, the proportion of paraneoplastic cases in our study (76%) is considerably higher than those previously reported (21%–66%).^3,5,9,11,27-32^ Nevertheless, nonparaneoplastic GABA_B_R-AE cases are scarcer in European descent populations^1-3,5,11,30,33^ than in Asian ones,^8,9,27-29,31,32,34,35^ which we also observed in our own series because all paraneoplastic cases with available DNA were of European descent and, conversely, almost 40% of nonparaneoplastic patients were not of European descent. This difference might reflect distinct HLA associations that we were unable to detect possibly due to the small sample size or might be related to other genetic loci or even environmental triggers (e.g., less smoking in Asian populations^31^); further immunogenetic research might be particularly revealing in non-European descent populations. In this study, patients classified as nonparaneoplastic underwent thorough and repeated tumor screening during an extended period of follow-up, except for 1 elderly patient who died early without treatment and for whom KCTD16-abs were positive but without any proof of cancer at autopsy (admittedly, a microscopic tumor could have been missed). Lower rates of paraneoplastic cases in previous studies could also reflect either methodological issues such as insufficiently extensive tumor screening or too short follow-ups.

The rate of relapse in our study (14%, similar between paraneoplastic and nonparaneoplastic cases) was also lower than previously described (19%–33%) in 3 other Asian series of patients with GABA_B_R-AE.^9,31,36^ However, only 3 of 166 patients (2%) in these studies were treated with second-line immunotherapy compared with ours (27%), which could explain the more frequent relapsing episodes and support the early use of second-line immunotherapies to prevent relapses; this has been recently suggested in other subtypes of AE.^37^ A long delay (more than 28 days) between disease onset and first-line immunotherapy was also hypothesized to raise the risk of relapse; however, the nonparaneoplastic group in our study evolved more favorably than the paraneoplastic group, even if on average they were treated later. The mortality rate found in our study (49%) was consistent with previously published studies from Northern countries (40–57%)^2,3,11^; the lower mortality found in some studies from Southern countries (23–42%)^8,9,31^ may be due to the greater frequency of nonparaneoplastic forms in Asian descent population.

Our study has some limitations, most of them being inherent to the retrospective methodology. Even if low in our study and not included in the analysis, missing data can still skew the results. To obtain the most homogeneous and comparable cohort, we deliberately excluded 3 patients with GABA_B_R-abs in serum because of discrepancies of immunologic testing, atypical clinical courses, and the co-occurrence of other neuronal antibodies that was believed to be more explanatory for the clinical presentation (Figure 1); this, however, may have led to omit a yet unknown neurologic phenotype of GABA_B_R-AE. Among the paraneoplastic group, 13 patients were included with a probable diagnosis of lung cancer, without reaching a tissue-based diagnosis of tumor; even with highly indicative clinical course and radiologic features, an erroneous diagnosis of cancer is still possible. Finally, the presence of cancer in the paraneoplastic group is also a major bias to compare the outcome and mortality because the cancer itself inevitably affects mortality of the paraneoplastic group. Finally, despite being helpful and easy to use, the choice of mRS as one of the outcome criteria might probably be insufficiently sensitive to detect clinically meaningful differences in outcome, especially in patients with prominent cognitive involvement.

This work also has some strengths, most importantly, all included cases were diagnosed or confirmed in a European Reference Network site for PNS, which uses in-house, highly reliable, diagnostic techniques.^38^ This European bicentric study is to our knowledge the largest Northern country cohort of GABA_B_R-AE.

To conclude, our study shows differences in demographic profile between paraneoplastic and some nonparaneoplastic GABA_B_R-AE cases, and suggests a different disease course in neurologic outcome between these 2 groups. The younger age as well as the more frequent use of second-line immunotherapy and the less common ICU admission of nonparaneoplastic patients may partly explain the latter result. We also confirmed the strong value of KCTD16-abs to identify paraneoplastic GABA_B_R-AE. In the light of these observations, disease mechanisms and immunogenetic features of paraneoplastic and nonparaneoplastic forms of GABA_B_R-AE should be further investigated to better understand these differences.

## Appendix Authors

**Table:**

Name	Location	Contribution
Florian Lamblin, MD	French Reference Center on Paraneoplastic Neurological Syndrome and Autoimmune Encephalitis, Hospices Civils de Lyon; Institut MeLiS INSERM U1314/CNRS UMR 5284, Université Claude Bernard Lyon 1; Department of Neurology, University Hospital of La Réunion, Saint-Pierre (La Réunion), France	Drafting/revision of the manuscript for content, including medical writing for content; major role in the acquisition of data; study concept or design; analysis or interpretation of data
Jeroen Kerstens, MD	Department of Neurology, Erasmus Medical Center, Rotterdam, The Netherlands	Drafting/revision of the manuscript for content, including medical writing for content; major role in the acquisition of data; analysis or interpretation of data
Sergio Muñiz-Castrillo, MD, PhD	Stanford Center for Sleep Sciences and Medicine, Stanford University, Palo Alto, CA	Drafting/revision of the manuscript for content, including medical writing for content; major role in the acquisition of data; analysis or interpretation of data
Alberto Vogrig, MD, PhD	Clinical Neurology, Department of Neurosciences, Azienda Sanitaria Universitaria Friuli Centrale (ASU FC); Department of Medicine (DAME), University of Udine Medical School, Udine, Italy	Major role in the acquisition of data
David Goncalves, PharmD	Department of Immunology, Hôpital Lyon Sud, Hospices Civils de Lyon, France	Major role in the acquisition of data
Veronique Rogemond, PhD	French Reference Center on Paraneoplastic Neurological Syndrome and Autoimmune Encephalitis, Hospices Civils de Lyon; Institut MeLiS INSERM U1314/CNRS UMR 5284, Université Claude Bernard Lyon 1, France	Major role in the acquisition of data
Geraldine Picard, MSc	French Reference Center on Paraneoplastic Neurological Syndrome and Autoimmune Encephalitis, Hospices Civils de Lyon; Institut MeLiS INSERM U1314/CNRS UMR 5284, Université Claude Bernard Lyon 1, France	Major role in the acquisition of data
Marine Villard, MSc	French Reference Center on Paraneoplastic Neurological Syndrome and Autoimmune Encephalitis, Hospices Civils de Lyon; Institut MeLiS INSERM U1314/CNRS UMR 5284, Université Claude Bernard Lyon 1, France	Major role in the acquisition of data
Anne-Laurie Pinto, MSc	French Reference Center on Paraneoplastic Neurological Syndrome and Autoimmune Encephalitis, Hospices Civils de Lyon; Institut MeLiS INSERM U1314/CNRS UMR 5284, Université Claude Bernard Lyon 1, France	Major role in the acquisition of data
Marleen H. Van Coevorden-Hameete, PhD	Department of Neurology, Erasmus Medical Center, Rotterdam, The Netherlands	Major role in the acquisition of data
Marienke A. De Bruijn, MD, PhD	Department of Neurology, Erasmus Medical Center, Rotterdam, The Netherlands	Major role in the acquisition of data
Juna M. De Vries, MD	Department of Neurology, Erasmus Medical Center, Rotterdam, The Netherlands	Major role in the acquisition of data
Marco Schreurs, MD	Department of Immunology, Laboratory Medical Immunology, Erasmus Medical Center, Rotterdam, The Netherlands	Major role in the acquisition of data
Louise Tyvaert, MD, PhD	Department of Neurology, University Hospital of Nancy, France	Major role in the acquisition of data
Lucie Hopes, MD	Department of Neurology, University Hospital of Nancy, France	Major role in the acquisition of data
Jerome Aupy, MD, PhD	Department of Clinical Neurosciences, University Hospital of Bordeaux, France	Major role in the acquisition of data
Cecile Marchal, MD	Department of Clinical Neurosciences, University Hospital of Bordeaux, France	Major role in the acquisition of data
Dimitri Psimaras, MD	Department of Neuro-Oncology, Pitié Salpêtrière Hospital, AP-HP, Paris, France	Major role in the acquisition of data
Laurent Kremer, MD	Department of Neurology, University Hospital of Strasbourg, France	Major role in the acquisition of data
Veronique Bourg, MD	Department of Neurology, Côte d'Azur University, Nice, France	Major role in the acquisition of data
Jean-Christophe G. Antoine, MD, PhD	Department of Neurology, University Hospital of Saint-Etienne, France	Major role in the acquisition of data
Adrien Wang, MD	Stroke Center Neurology Division, Hopital Foch, Suresnes, France	Major role in the acquisition of data
Philippe Kahane, MD, PhD	University Grenoble Alpes, Inserm, U1216, CHU Grenoble Alpes, Grenoble Institut Neurosciences, France	Major role in the acquisition of data
Sophie Demeret, MD, PhD	Neurological Intensive Care Unit, Pitié-Salpêtrière Hospital, AP-HP, Paris, France	Major role in the acquisition of data
Guido Ahle, MD	Department of Neurology, Hôpitaux Civils de Colmar, France	Major role in the acquisition of data
Vicente Peris Sempere, MSc	Stanford Center for Sleep Sciences and Medicine, Stanford University, Palo Alto, CA	Analysis or interpretation of data
Noemie Timestit, MSc	Department of Public Health, Hospices Civils de Lyon, France	Analysis or interpretation of data
Mikail Nourredine, MD	Department of Public Health, Hospices Civils de Lyon, France	Analysis or interpretation of data
Aurelien Maureille, MD	Department of Medicine, Centre Leon Berard, UNICANCER, Lyon, France	Major role in the acquisition of data
Marie Benaiteau, MD	French Reference Center on Paraneoplastic Neurological Syndrome and Autoimmune Encephalitis, Hospices Civils de Lyon; Institut MeLiS INSERM U1314/CNRS UMR 5284, Université Claude Bernard Lyon 1, France	Drafting/revision of the manuscript for content, including medical writing for content
Bastien Joubert, MD, PhD	French Reference Center on Paraneoplastic Neurological Syndrome and Autoimmune Encephalitis, Hospices Civils de Lyon; Institut MeLiS INSERM U1314/CNRS UMR 5284, Université Claude Bernard Lyon 1, France	Drafting/revision of the manuscript for content, including medical writing for content; analysis or interpretation of data
Emmanuel Mignot, MD, PhD	Stanford Center for Sleep Sciences and Medicine, Stanford University, Palo Alto, CA	Drafting/revision of the manuscript for content, including medical writing for content; analysis or interpretation of data
Maarten J. Titulaer, MD, PhD	Department of Neurology, Erasmus Medical Center, Rotterdam, The Netherlands	Drafting/revision of the manuscript for content, including medical writing for content; study concept or design; analysis or interpretation of data
Jerome Honnorat, MD, PhD	French Reference Center on Paraneoplastic Neurological Syndrome and Autoimmune Encephalitis, Hospices Civils de Lyon; Institut MeLiS INSERM U1314/CNRS UMR 5284, Université Claude Bernard Lyon 1, France	Drafting/revision of the manuscript for content, including medical writing for content; study concept or design; analysis or interpretation of data

## Glossary

AE: autoimmune encephalitis
CBA: cell-based assay
GABA_B_R-AE: gamma-aminobutyric-acid B receptor antibodies
GWAS: genome-wide association data
HLA: human leukocyte antigen
ICU: intensive care unit
IgG: immunoglobulin G
IQR: interquartile range
LE: limbic encephalitis
PNS: paraneoplastic neurologic syndromes
SCLC: small cell lung cancer
